# Evaluation of a High Concentrate Omega-3 for Correcting the Omega-3 Fatty Acid Nutritional Deficiency in Non-Alcoholic Fatty Liver Disease (CONDIN)

**DOI:** 10.3390/nu10081126

**Published:** 2018-08-20

**Authors:** Derek Tobin, Merethe Brevik-Andersen, Yan Qin, Jacqueline K. Innes, Philip C. Calder

**Affiliations:** 1 BASF AS, NO-1327 Lysaker, Norway; Yan.Qin@basf.com; 2 Formerly BASF AS, NO-1327 Lysaker, Norway; merethha@gmail.com; 3 Human Development and Health Academic Unit, Faculty of Medicine, University of Southampton, Southampton SO16 6YD, UK; innesjackie@gmail.com (J.K.I.); P.C.Calder@soton.ac.uk (P.C.C.); 4 National Institute for Health Research Southampton Biomedical Research Centre, University Hospital Southampton NHS Foundation Trust and University of Southampton, Southampton SO16 6YD, UK

**Keywords:** non-alcoholic fatty liver disease, NAFLD, omega-3 fatty acid, EPA, DHA, omega-3 index

## Abstract

This randomized controlled trial investigated the safety and efficacy of MF4637, a high concentrate omega-3 fatty acid preparation, in correcting the omega-3 fatty acid nutritional deficiency in non-alcoholic fatty liver disease (NAFLD). The primary end point of the study was set as the change of red blood cell (RBC) eicosapentaenoic acid (EPA) and docosahexaenoic acid (DHA) by MF4637. Whether the omega-3 concentrate could lower liver fat was evaluated in a subset of patients. Furthermore, 176 subjects with NAFLD were randomized to receive the omega-3 concentrate (*n* = 87) or placebo (*n* = 89) for 24 weeks, in addition to following standard-of-care dietary guidelines. The omega-3 index, omega-6: omega-3 fatty acid ratio and quantitative measurements of RBC EPA and DHA were determined at baseline and study completion. Magnetic resonance imaging of liver fat was conducted in a subset of patients. Administration of high concentrate omega-3 for 24 weeks significantly increased the omega-3 index and absolute values of RBC EPA and DHA, and decreased the RBC omega-6: omega-3 fatty acid ratio (*p* < 0.0001). A significant reduction in liver fat content was reported in both groups.

## 1. Introduction

Non-alcoholic fatty liver disease (NAFLD) is the presence of hepatic steatosis (>5% liver fat assessed by imaging modalities or >5% of cells containing visible lipid droplets from histology) that is not related to significant alcohol consumption, hereditary disorders, viral infection or steatogenic medication [1]. Early NAFLD is typically reversible, but can develop in some 30% of cases into non-alcoholic steatohepatitis (NASH), presenting as hepatic steatosis with inflammation, ballooning and evidence of hepatocellular injury with or without fibrosis [1,2,3]. NAFLD is associated with metabolic risk factors such as obesity, diabetes and dyslipidemia, and its prevalence has risen sharply in line with the rising rates of obesity and diabetes [1,4,5]. In Western countries, NAFLD is the leading cause of liver disease [6]. NAFLD is estimated to affect 20–30% of the general population, with the prevalence increasing to approximately 75% of patients with obesity or diabetes, and 90–95% in the morbidly obese [6,7,8,9]. The estimated prevalence of NASH is lower, but significant, at 2–3% of the general population and one-third of the morbidly obese [1,7].

Identification of NAFLD patients in a clinical setting is commonly performed due to suspicion from raised liver enzymes and confirmation of hepatic steatosis by ultrasound. More recently, more advanced techniques such as Fibroscan (Echosens) and lipidomic analysis (OWL) have become available at specialised and general practitioner level. A validated algorithm for steatosis risk, called the fatty liver index (FLI), uses clinically available measurements to predict steatosis and to identify populations at risk of developing further liver-related morbidities [10].

NAFLD is increasingly considered as the hepatic manifestation of metabolic syndrome. The metabolic dysregulation occurring during metabolic syndrome often starts with excess peripheral fat and peripheral insulin resistance with hepatic steatosis and hepatic insulin resistance following as secondary events. Hepatic insulin resistance results in increased circulating glucose and very low density lipoproteins (VLDLs) [11]. The resulting hyperglycemia, hyper-triglyceridaemia and lowered HDL-cholesterol are all risk factors for the development of cardiovascular disease (CVD) [1,2,11,12]. In common with metabolic syndrome, NAFLD is associated with increased morbidity and mortality, particularly from CVD [1,2,12,13]. NASH also has the potential to develop into liver cirrhosis, from which 30–40% of patients will die of liver-related causes such as liver failure or hepatocellular carcinoma within a ten-year period [2,3,14]. Despite the increasing prevalence of NAFLD and its associated morbidity and mortality, there is currently no approved drug therapy for its treatment. The World Gastroenterology Organization (WGO) guidelines state that, in addition to pharmacological management of co-morbidities such as diabetes and dyslipidemia, weight loss and increased physical exercise are the most effective ways to reduce liver fat [6]. However, such lifestyle changes are typically difficult to sustain in the long term, creating a significant unmet need for this condition.

There is mounting evidence that long-chain polyunsaturated fatty acids (PUFAs), especially the marine omega-3 fatty acids eicosapentaenoic acid (EPA) and docosahexaenoic acid (DHA), are depleted in patients with NAFLD [15,16,17,18,19]. This may be due to several factors including impairment of the hepatic metabolic pathways responsible for the synthesis of EPA and DHA from their precursors, increased utilization due to lipid peroxidation caused by raised oxidative stress in NALFD, as well as reduced dietary intake [15,16,20,21]. Increased levels of omega-3 PUFAs and reduction of the omega-6: omega-3 ratio enables a shift in hepatic fat metabolism away from de novo lipogenesis and towards fatty acid oxidation and secretion, thereby potentially reducing steatosis in NAFLD [17,22,23,24,25,26]. In support of this, a recently published systematic review and meta-analysis of omega-3 fatty acids in NAFLD patients demonstrate statistically and clinically significant consistent reduction in steatosis with approximately 3 g EPA plus DHA daily [27]. Overall, existing data demonstrate that NAFLD patients have reduced levels of EPA and DHA compared to healthy individuals and that there are beneficial effects on liver steatosis from increased intake of omega-3 PUFAs at approximately 3 g/day.

The purpose of this study was to investigate the safety and the ability of an omega-3 fatty acid medical food (MF4637; BASF AS, Lysaker, Norway), to correct the omega-3 fatty acid nutritional deficiency present in NAFLD. The hypotheses being tested are that MF4637 will significantly improve the omega-3 index (EPA + DHA in red blood cells (RBCs)) and lower the RBC omega-6: omega-3 fatty acid ratio in patients with NAFLD. The potential for MF4637 to reduce hepatic fat content was evaluated using magnetic resonance imaging-proton density fat fraction (MRI-PDFF) in a subset of patients. Additional post hoc stratification was performed using the FLI.

## 2. Materials and Methods

### 2.1. Study Design

This was a randomized double-blind placebo-controlled study conducted at 21 investigative sites across the U.S. All procedures involving human participants were approved by Quorum Review IRB, Seattle, WA, USA. All participants provided written informed consent. The trial is registered with ID NCT02923804 at the U.S. National Library of Medicine’s ClinicalTrials.gov website.

**Figure 1:**
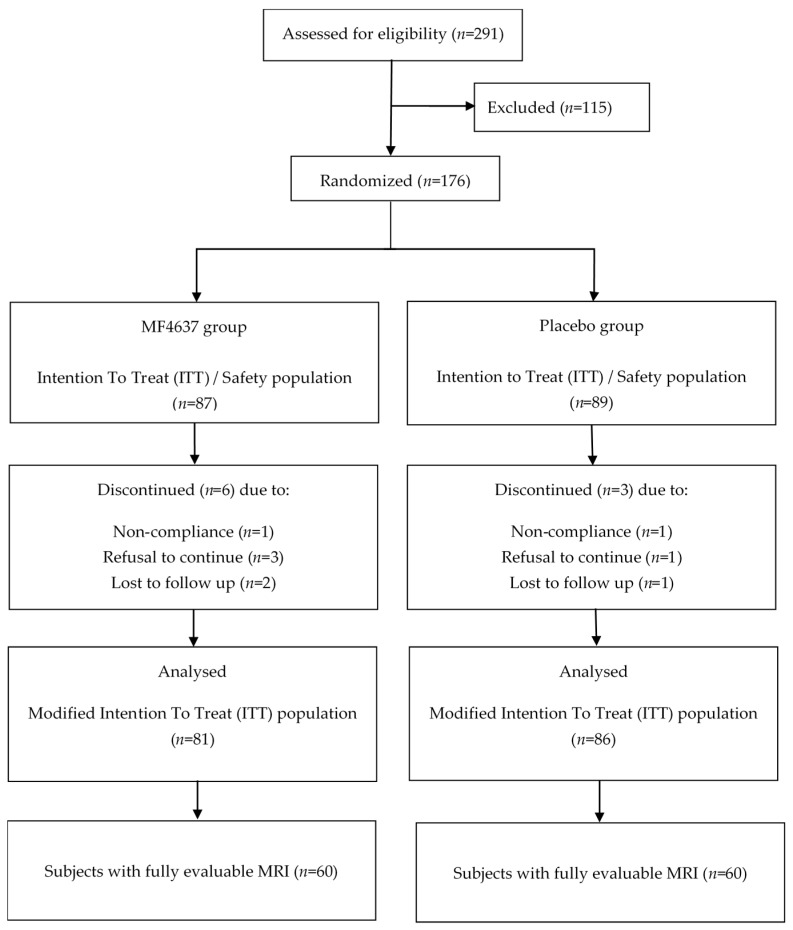
CONSORT flow chart of participant flow.

Participants were recruited based on the suspected diagnosis of NAFLD, confirmed either by diagnostic imaging performed within the previous year, or by abdominal ultrasound performed at screening. Eligibility was determined, after the informed consent process, at screening, which included review of medical history and current medications, measurement of vital signs (height, weight, blood pressure, heart rate and body mass index (BMI)), hemoglobin A1c (HbA1c), thyroid-stimulating hormone (TSH) and liver function testing. Following written informed consent, each participant was centrally randomized 1:1, stratifying by site, marine omega-3 fatty acid intake (≥250 mg/day and <250 mg/day), diabetes and statin use, to receive either the omega-3 concentrate MF4637 or a placebo (olive oil) for 24 weeks. The duration of the study was determined by taking into account the delay of approximately 6 months required for stabilization of the incorporation of EPA and DHA demonstrated in healthy subjects [28] and an analysis of the literature on the use of omega-3 during NAFLD for periods ranging from 6 months to more than one year [27]. Randomization numbers corresponding to predetermined intervention were assigned in a sequential manner to each subject via an Interactive Voice/Web Response System. One hundred and seventy-six subjects were subsequently randomized to receive either MF4637 (*n* = 87) or placebo (*n* = 89) (Figure 1). The modified intention to treat population was defined as all subjects who took at least a 1-day dose of omega-3 fatty acids or placebo and underwent at least 1 post-randomization primary efficacy assessment.

The omega-3 fatty acid medical food (MF4637; BASF AS, Lysaker, Norway) was provided as soft gel capsules, with each 1 g capsule containing marine-sourced EPA and DHA as ethyl esters (460 mg and 380 mg, respectively). Placebo capsules were identical in size and appearance to MF4637 and contained 1 g of olive oil. The investigational products were administered in a double-blinded fashion. Study participants were required to take three capsules per day of either MF4637 or placebo with food for 24 weeks. Thus, daily intakes of EPA and DHA in the MF4637 group were 1.38 g and 1.14 g, respectively. Compliance was measured via subject interview and unused capsule counts.

In addition to the investigational product, study participants were advised to reduce normal caloric intake as recommended by the American Association for the Study of Liver Disease (AASLD) standard-of-care guidelines for NAFLD [1], and to maintain stable physical activity levels throughout the study. To provide the American Heart Association (AHA) recommended dietary intake of omega-3 fatty acids [29], participants were required to consume two meals of omega-3 rich fish per week (from a choice of salmon, herring, whitefish, sardines, bluefish and trout) and to reduce foods rich in trans- and omega-6 fatty acids (fried foods and snacks, fast foods, bacon, turkey bacon, hams, nuts, peanut butter, sesame seeds, sunflower seeds, pumpkin seeds, vegetable oils and margarine (including soybean oil and corn oil), mayonnaise and salad dressing). Dietary intake was monitored regularly throughout the study via participant’s food diaries.

At baseline (week 0), week 12 and study completion (week 24), weight, blood pressure, heart rate and BMI were recorded and blood samples collected to assess efficacy (Omega-3 index, RBC omega-6:omega-3 ratio and quantitative measurements of RBC EPA and DHA) and safety (vital signs, standard clinical biochemistry and hematology panels including liver function tests). Adverse events were monitored throughout the study. MRI-PDFF assessments of liver fat were performed at baseline (week 0) and study completion (week 24).

The primary endpoint of the trial was to test the effect of administration with concentrated EPA and DHA on the omega-3 index (RBC EPA + DHA). Secondary endpoints included quantitative measurement of RBC EPA and DHA and assessment of the RBC omega-6: omega-3 ratio. The potential for MF4637 to reduce hepatic fat content was evaluated as an exploratory outcome.

### 2.2. Inclusion and Exclusion Criteria

Selection of the NAFLD study population aimed to include subjects with hepatic steatosis, excluding those with a known previous diagnosis, at any time, of NASH indicating more advanced liver disease. Liver biopsy and histopathology are the only secure means of differentiating NASH from NAFL and therefore the study population may contain NASH patients. Inclusion criteria included age ≥18 years and a recent (<1 year) suspected clinical diagnosis of NAFLD including an imaging modality (e.g., ultrasound). If diagnosis was >1 year or an imaging test was absent, an abdominal ultrasound was performed at screening to confirm diagnosis of NAFLD. Other inclusion criteria included not smoking, BMI between 18–39.9 kg/m^2^ and, if on statin medication, a history of >1 month on a stable dose. Exclusion criteria included a diagnosis of NASH; bilirubin >2 times the upper limit of normal; other causes of liver inflammation i.e., hepatitis A, B or C, HIV, cirrhosis, Wilson’s disease, autoimmune hepatitis, hemochromatosis, alcoholic steatohepatitis, pancreatitis, or prescription medications known to cause liver toxicity or damage; history of bariatric surgery; significant weight loss (>5% body weight) or rapid weight loss (>1.6 kg/week) within six months of screening; cancer; significant cardiovascular disease including untreated hypertension and significant gastrointestinal, renal, pulmonary, hepatic, biliary or endocrine disease. Furthermore, subjects were excluded if there was significant alcohol consumption; use of any medicine or dietary supplement that may affect NAFLD or lipid metabolism (including omega-3 supplements); use of anti-coagulants; and pregnancy/breastfeeding or sensitivity to any of the study medications or excipients.

### 2.3. Measurement of RBC EPA and DHA

#### 2.3.1. Quantitative Measurement of RBC EPA and DHA

Concentrations of total RBC EPA and DHA were measured quantitatively using UPLC-MS/MS. Method validation and all measurements were performed by Diteba Analytical and Bioanalytical Services, A Nutrasource Company (Mississauga, ON, Canada). Blood samples were collected into EDTA vacationer tubes, centrifuged, and plasma and white blood cells (buffy coat) removed. The remaining RBCs were washed three times with saline, and 0.5 mL of the washed packed RBCs added to 1 mL of distilled water, to which was added 150 µL of EDTA/ascorbic acid. The sample was mixed well and stored at −80 °C until analyzed.

For the quantitative analytical methodology, a specific amount of standard curve solutions, matrix blanks, quality control samples and thawed study samples were acidified with HCl and internal standard was added to all tubes except for blanks. Samples were mixed well and incubated at 100 °C for 45 min, and then cooled to room temperature. Extraction solvent (hexane: dichloromethane: 2-propanol, in a 20:10:1 ratio) was added to each tube, which was mixed well and centrifuged. Capped tubes were submerged in a dry ice-acetone bath to freeze the aqueous layer, and the organic layer in each tube was transferred to another tube. This was evaporated to dryness at 45 °C; then, reconstitution solution was added, mixed thoroughly, and reconstituted samples were transferred into LC-MS vials for injection. The UPLC-MS/MS systems consisted of an Aquity Tandem Quadruple detector, auto-sample manager, binary solvent manager, column manager and Empower 3 data acquisition system. The UPLC column for optimum chromatographic conditions was an Aquity UPLC BEH, C18, 2.1 mm × 50 mm, 1.7 µm, assembled with a Waters in-line pre-column filter. The mobile phase was a 20:80 mixture of 5 mM ammonium acetate in water and acetonitrile. The injection volume was 5.0 mL, flow rate was 0.30 mL/min, run time was approximately 2.5 min, column temperature was ambient and sample temperature was 5 °C ± 2 °C. From the resulting chromatograms, EPA and DHA in each sample were calculated by calibration curve using peak area response ratio as response function. The quantitative method provided a range of 1 to 500 µg/mL for EPA, and 5 to 500 µg/mL for DHA.

#### 2.3.2. Qualitative Measurement of RBC EPA and DHA

Qualitative measurement of EPA and DHA involved measuring the fatty acid profile of RBCs (consisting of a total of 30 fatty acids) using a gas chromatograph system with an auto sampler and FID detector (Diteba Analytical and Bioanalytical Services, Guelph, ON, Canada). Blood sample collection and processing was identical to that of quantitative analysis of RBC EPA and DHA. A specified amount (2 mL) of BF_3_-MeOH was added to thawed RBC samples, mixed, flushed with N_2_ gas and incubated for 10 min at 100 °C. After cooling, 250 µL of purified water and 750 µL of heptane were added to each tube and mixed well. Tubes were centrifuged at 4,000 rpm for 5 min and the top heptane layer was transferred to another tube and washed with purified water. The top (heptane) layer was transferred to another tube and evaporated to dryness under a stream of N_2_ gas at 50 °C. Each tube was reconstituted with 10 µL of heptane, transferred to a GC vial and flushed with N_2_ in preparation for injection. For this methodology, the column was a DB Wax, 30 m × 0.25 mm ID, 0.15 µm film or equivalent. The chromatic conditions were a GC with an FID detector, helium carrier gas, an initial oven temperature of 170 °C, increased at 3 °C/min to 200 °C, held for 3 min, increased at 2.5 °C/min to 225 °C, held for 5 min, then increased at 20 °C to 245 °C, and then held for 12 min. An external standard was injected three times and then reinjected for every 10 sample injections. From the three consecutive standard injections, an average response factor (RF) for each individual fatty acid was calculated, using the peak area of each individual fatty acid detected.

### 2.4. Assessment of Change in Liver Fat

Assessment of the change in hepatic fat fraction was measured via MRI-PDFF. For each subject, the MRI protocol included a localization sequence and a two-dimensional six-echo spoiled gradient-recalled-echo breath hold sequence. A three-plane localizer followed by a coronal breath-hold localizer was recommended for accurate axial slice prescription. If the scanner was not capable of acquiring six echoes simultaneously, multiple acquisitions with single-echo sequences were performed. From either the six-echo or six single-echo MRI series, the radiologist identified a circular region of interest (ROI) within each of the nine Cournand segments of the liver using the first echo of the series. The radiologist then identified regions with an approximately 2.5 cm diameter in each of the nine Cournand segments, except for segment 1 (the caudate), in which a region with a diameter of approximately 1.5 cm was identified. The ROI in the caudate was smaller since the caudate is generally too small to identify a region larger than 1.5 cm. The radiologist excluded blood vessels and the periphery of the liver when identifying the ROIs. A fat fraction map was calculated from the six-echo sequence using a multi-interference technique, which took into account the contribution from the individual resonances in the fat spectrum to the observed MRI signal to obtain an accurate estimate of fat. The whole liver hepatic fat fraction (HFF) was expressed as the mean fat fraction across all 9 user-defined ROIs in the liver.

### 2.5. Calculation of Fatty Liver Index (FLI) Score

Fatty liver index (FLI), an algorithm used to predict the presence of hepatic steatosis based on measured values for serum triglycerides (in mg/dL), serum GGT (in IU/L), BMI (in kg/m^2^) and waist circumference (in cm), was calculated using the following equation [10]:

FLI = (e 0.953 × loge (triglycerides) + 0.139 × BMI + 0.718 × loge (GGT) + 0.053 × waist circumference-15.745)/(1 + e 0.953 × loge (triglycerides) + 0.139 × BMI + 0.718 × loge (GGT) + 0.053 × waist circumference-15.745) × 100.
(1)

### 2.6. Sample Size Calculations

It has been reported that the omega-3 index is 0.5% higher in healthy subjects compared to those with some form of liver dysfunction, leading to the assumption that a minimum increase of 0.5% in RBC EPA + DHA may be necessary to achieve nutritional sufficiency in NAFLD patients [30,31]. A conservative between-intervention difference for RBC EPA + DHA, measured as a change from baseline score between standard of care and standard of care plus MF4637, was set at 1.0% [32] and the standard deviation at 2.0 with a correlation of 0.5 and equal allocation of subjects across the two intervention groups, yielding 64 subjects per intervention arm for a total of 128. To address the uncertainty of the estimates of intervention effectiveness from the emerging literature, an adaptive blinded mid-course sample size re-estimation procedure was originally planned for the point at which approximately 30% of the subjects had completed one post-baseline visit (i.e., to Week 12) and had provided the RBC EPA + DHA results (for baseline and Week 12). The sample size re-estimation was performed by one unblinded study statistician. When 30% of the subjects had provided the Week 24 RBC DHA and EPA data, and the data were considered “lockable” by data management, the data file was exported to a limited access subdirectory, the effect size (change from baseline in plasma level) estimated and the conditional power (CP) were calculated. Because the CP was between 41% and 90%, the number of subjects per intervention arm was increased, in order to recover the targeted power of 90%. Given that the interim analysis was performed at 30% of the initial sample size, and the targeted power was 90%, the minimum conditional power cut-off value (CP min) was set at 41%. The procedure was performed, as per the Charter, and the recommendation was to increase the sample size to 75 subjects per intervention (i.e., 150 subjects). The actual number of participants recruited to the study was 176.

### 2.7. Statistical Analysis

The primary outcome (RBC EPA + DHA) was analyzed using a repeated analysis of covariance (ANCOVA) with the stratification factors as covariates, in order to compare the changes in the combined EPA + DHA outcome between the two intervention groups (MF4637 group and placebo) across the study. Stratification factors were baseline omega-3 intake, diabetes status and statin use. Additional outcomes were analyzed using the same ANCOVA model applied to the primary outcome: RBC EPA, RBC DHA and the omega-6: omega-3 ratio. All programming was performed in SAS version 9.2 (SAS Institute, Cary, NC, USA) or higher under the Windows Server 2008R2 operating system.

A regression analysis for absolute RBC EPA + DHA and change in liver fat for the MF4637 group and placebo was planned without testing of statistical significance. Similarly, regression analysis of changes in omega-3 index against baseline omega-3 index was performed. Post hoc analysis of the change in liver fat for intervention and placebo groups after stratification using the baseline Fatty Liver Index was tested using ANCOVA.

## 3. Results

Of the 176 participants that underwent randomization, safety was assessed for 87 subjects in the intervention group and 89 in the placebo group (Figure 1). Of those participants randomized, 167 (81 in the MF4637 group and 86 in the placebo group) were included in the modified intention to treat (mITT) primary outcome analysis.

Baseline anthropometric and biochemical variables of participants randomized to the placebo and MF4637 groups are detailed in Table 1. Of note is the higher mean fasting insulin concentration in the placebo group, which, together with the comparable mean fasting glucose concentration, suggests a likelihood that the placebo group was more insulin resistant than the MF4637 group at study entry.

Table 2 details the main anthropometric and biochemical variables for participants randomized to the placebo and MF4637 groups at baseline and after 24 weeks of intervention. The overall weight of subjects in either intervention group remained unchanged from study start to study end. Triglyceride levels at baseline were similar in both groups and would be clinically regarded as borderline raised. At the end of the study, a statistically significant reduction in triglycerides was only seen in the MF4637 group. Interestingly, the liver enzymes ALT, AST and GGT showed statistical reductions at the end of study in the placebo group and not in the MF4637 group. This implies an overall improvement in liver function without weight loss.

Compliance regarding the investigational products was 89% in the MF4637 group and 91% in the placebo group. There were no serious adverse events related to study interventions reported during the 24-week study. Mild incidences of eructation (*n* = 1), dysgeusia (*n* = 1), abdominal bloating (*n* = 1) and increased blood triglycerides (*n* = 1) together with a moderate case of diarrhea (*n* = 1) were reported in the MF4637 group and suspected to be related. One participant in the MF4637 group and two participants in the placebo group discontinued the study due to adverse events.

### 3.1. Effect of Intervention on Omega-3 Index

The baseline omega-3 index was similar for placebo and intervention groups. Compared to placebo, the mean omega-3 index increased significantly from 4.8% to 8.0% at study completion in the MF4637 group, representing a mean 3.2% change from baseline (*p* < 0.0001) (Table 3). In the placebo group, the omega-3 index increased slightly from 4.9% at baseline to 5.3% at study completion, representing a mean change of 0.4%. Regression analysis of the data for participants in the MF4637 group suggests that the change in omega-3 index was inversely related to the baseline omega-3 index, with lower baseline values resulting in greater increases by the end of the 24-week intervention (data not shown [33]).

### 3.2. Effect of Intervention on RBC EPA + DHA, EPA and DHA Values

Absolute RBC EPA + DHA increased on average from 29.6 µg/mL at baseline to 52.9 µg/mL at study completion (representing a significant increase of 21.2 µg/mL) in the MF4637 group, compared to a 1.2 µg/mL increase from baseline in the placebo group (significance between groups, *p* < 0.0001) (Table 3). In terms of absolute values of EPA and DHA separately, RBC EPA increased by a significant 7.1 µg/mL to 10.6 µg/mL at study completion in the MF4637 group versus a 0.4 µg/mL increase in the placebo group (*p* < 0.0001) (Table 3). RBC DHA increased by more than EPA, with a mean of 14.1 µg/mL increase to 42.4 µg/mL in the MF4637 group versus a 0.7 µg/mL increase in the placebo group (*p* < 0.0001) (Table 3).

Regarding the percentage of individual EPA and DHA as a proportion of total RBC fatty acids, both parameters increased significantly in the MF4637 group compared to placebo (Table 3). Specifically, RBC EPA as a percentage of total fatty acids increased by a significant 0.9% to 1.4% in the MF4637 group compared to a 0.002% increase in the placebo group (*p* < 0.0001). RBC DHA as a percentage of total fatty acids increased by a greater proportion than EPA, resulting in a 2.3% increase to 6.6% in the MF4637 group versus 0.4% increase in the placebo group (*p* < 0.0001).

### 3.3. Effect of Intervention on RBC Omega-6: Omega-3 Ratio

RBC omega-6: omega-3 ratio was not different between the two groups at baseline. Following administration of MF4637 for 24 weeks, the RBC omega-6: omega-3 ratio decreased by a mean of 1.6 from 4.9 at baseline to 3.3 at study completion, compared to a 0.2 decrease to 4.7 in the placebo group (*p* < 0.0001) (Table 3).

### 3.4. Effect of Intervention on Liver Fat

In the mITT analysis of liver fat content, 120 participants (60 in each trial arm) completed both the baseline and end of study MRI-PDFF assessment. In this population, baseline liver fat was 17.4% in the placebo group and 14.4% in the MF4637 group (*p* = 0.0689). Both the MF4637 and placebo groups demonstrated a decrease in liver fat percentage (26% and 28%, respectively), (Table 4). As such, there was no statistically significant difference in the decrease in liver fat between the groups.

### 3.5. Relationship between RBC EPA + DHA Enrichment and Liver Fat Content

**Figure 2:**
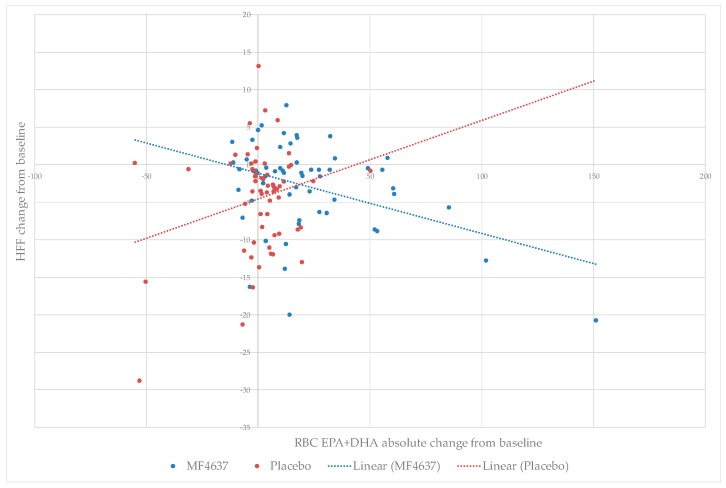
Relationship between change in absolute RBC EPA + DHA and change in liver fat.

Regression analysis of the data by intervention group suggests that the change from baseline in liver fat percentage was inversely related to the change in absolute RBC EPA + DHA values in the MF4637 group. Thus, the largest decreases in liver fat were observed in participants with the greatest increases in absolute RBC EPA + DHA (Figure 2). Hence, whilst there was no significant difference between MF4637 and placebo with regard to overall reduction of liver fat, there was an association between increasing RBC EPA+DHA enrichment and decreasing percentage liver fat content.

### 3.6. Relationship between Baseline Fatty Liver Index (FLI) and Change in Liver Fat Content

Post hoc analysis of the MF4637 group utilizing ANCOVA with baseline fatty liver index (FLI) as covariate found that, in those patients with higher baseline FLI scores (indicative of more probable fatty liver), there was a greater reduction in liver fat compared to placebo (Table 5). Following 24 weeks of intervention with MF4637, patients with baseline FLI ≥40 (*n* = 17) had a placebo corrected, statistically significant 44% relative decrease in liver fat content (*p* = 0.009). This equates to a 7.45% absolute decrease in placebo corrected liver fat content for the MF4637 group.

### 3.7. Relationship between RBC EPA + DHA Enrichment and Liver Enzymes AST and ALT

At study entry, the mean baseline concentrations of the liver enzymes AST and ALT were within the normal range for both the placebo and MF4637 groups. This is not surprising considering that liver enzymes may be normal in up to 80% of NAFLD patients [34]. Similar to the relationship between RBC EPA + DHA enrichment and change in liver fat discussed above, a non-statistical inverse association was also found between the change in absolute RBC EPA + DHA and change in the concentrations of the liver enzymes AST and ALT in the intervention group. Thus, with increasing change in absolute RBC EPA + DHA, there were greater decreases in both AST and ALT concentrations (data not shown). However, there were significant reductions in AST, ALT and GGT in the placebo group (unrelated to omega-3 measurements), suggesting that multiple factors may be impacting the clinical outcome of the study groups.

### 3.8. Effect of Intervention on Plasma Triglycerides

At study completion, plasma triglycerides (Table 2) decreased by a statistically significant 18% from baseline values in the intervention group (*p* = 0.0008) compared to a 7% reduction in triglycerides from baseline values in the placebo group (*p* = 0.52; for placebo adjusted effect of M4637 *p* = 0.053). The baseline levels for triglycerides were only moderately increased compared to normality and would clinically be defined as “borderline high”.

## 4. Discussion

This study demonstrates that intervention with high concentrate omega-3 for 24 weeks significantly raises the omega-3 index and decreases the omega-6: omega-3 fatty acid ratio in adults with NAFLD. Furthermore, the EPA and DHA enrichment achieved with intervention was significantly greater than that obtained by dietary recommendation alone. This is of importance, considering the depleted omega-3 status of NAFLD patients [15,16,17,18,19] and the current lack of therapeutic options for the treatment of NAFLD other than lifestyle recommendation [6]. Furthermore, the metabolic efficacy of the high concentrate omega-3 was confirmed through its significant lowering of plasma triglyceride levels compared to baseline levels.

When assessing the mITT population from whom baseline and post-intervention data from MRI-PDFF were available, intervention with both a high concentrate omega-3 and placebo caused a significant reduction in hepatic steatosis that was significant within each of the groups. There may be several reasons for the liver fat-lowering effect observed in the placebo group. All study participants were required to follow the standard-of-care dietary recommendations for the management of NAFLD. This included adherence to a diet with reduced caloric intake, and increased omega-3 and reduced omega-6 and trans-fatty acid consumption. Hence, participants in the placebo group may have achieved a decrease in liver fat percentage from the effects of these dietary recommendations alone, particularly from increased omega-3 fatty acid intake from the diet. However, it should be remembered that the intervention group had a greater increase in omega-3 index than placebo, suggesting a minimal influence from dietary changes. Similar findings of liver fat improvement in the placebo group have been reported in several other studies. These studies propose that MRI-PDFF volatility in early NAFLD subjects may contribute to data variability [35].

Of general note in the current study is the relatively low baseline liver fat by MRI-PDFF (mean 17% and 14% in placebo and intervention groups, respectively), which, together with relatively low baseline AST and ALT levels, indicate an early stage of NAFLD in this study population. Early stages of NAFLD are characterized by changeable liver fat content that can be affected by factors such as high-fat meals. This is in contrast to advanced NAFLD, in which the liver fat is likely to be more stable and less influenced by such factors.

A further confounding factor may be the high number (over one-third) of diabetic participants, and the number of subjects taking metformin and thiazolidinedione during the trial. From the mean baseline fasting insulin and glucose concentrations, the placebo group is also likely to have been more insulin resistant than the intervention group at study entry. These factors may have had some effect on liver fat metabolism. Indeed, on stratification of the data by diabetes status, those with diabetes had a greater reduction in liver fat from baseline (mean decrease of 4.9% in MF4637 group versus 6.3% decrease in placebo group) compared to those that did not have diabetes (mean decrease of 1.6% in MF4637 group versus 3.3% decrease in placebo group). To date, there have been very few trials conducted in the diabetic NAFLD population. One limitation of this study is the lack of additional lifestyle background information on variables that may act as confounders; these include smoking habits, annual income, academic background, and level of physical activity both at baseline and at the end of the study.

A number of individual studies and several meta-analyses have reported favorable outcomes with omega-3 fatty acid intervention in patients with NAFLD [27,36,37]. Despite a high degree of heterogeneity in patient population, study duration, dose and form of omega-3 fatty acids, a recent meta-analysis concluded that omega-3 fatty acids are associated with significant improvements in liver fat content and the liver enzymes ALT and GGT when taking approximately 3 g/day of EPA and DHA [27]. The positive effect of omega-3 fatty acids on liver fat was also confirmed in an earlier meta-analysis [37]. Surprisingly, only four of the eight trials performed to date included some form of measurement of EPA and DHA enrichment following intervention [38,39,40,41]. A strength of the current study is the measurement of both omega-3 fatty acids and omega-6: omega-3 fatty acid ratio, as well as the quantification of individual EPA and DHA in RBCs at baseline and study completion. This has enabled additional regression analyses to be performed, which suggest an inverse relationship between change in absolute RBC EPA + DHA and change in liver fat content and liver enzyme concentrations, although these data are exploratory and not supported by statistical analysis. Similar findings were reported in a study of high dose omega-3 in NAFLD patients where beneficial effects on liver fat content correlated with DHA content in RBCs [40]. Another strength of the current study was the use of MRI-PDFF to accurately assess change in liver fat content, which is the most accurate assessment method besides highly invasive liver biopsy [42].

A limitation of this study was the finding of a relatively low level of hepatic steatosis in participants, which restricted the potential for more significant effects to be observed on liver-related outcomes.

Post hoc use of the FLI to stratify patients showed an association between higher FLI scores and greater decrease in hepatic fat in the omega-3 intervention group. FLI scores below 30 are predictive of a liver without steatosis [10]. The high number of subjects with FLI < 30 confirms that this study recruited a relatively healthy population. However, highly statistically significant improvements in hepatic fat content were seen in those with a baseline FLI > 40, suggesting that this patient group can receive beneficial effects of intervention compared to placebo. Such a use of FLI is in accordance with the aims of its developers who propose that the “potential clinical uses of FLI include the selection of subjects to be referred for ultrasonography and the identification of (NAFLD) patients for intensified lifestyle counselling” [10]. Stratification by MRI-PDFF assessed steatosis levels was unable to identify omega-3 “responders”; however, the FLI score was able to do this. In this case, we can consider the study population as a population enriched for the presence of hepatic steatosis. The FLI score is composed, in part, of measurements of triglycerides and the liver enzyme GGT. Increased triglycerides in the liver is the cause of steatosis and increased liver enzymes are a consequence of liver damage. Plasma triglycerides are sensitive to omega-3 fatty acid intervention. In a meta-analysis, omega-3 fatty acids were shown to decrease liver enzymes (in particular GGT) in NAFLD patients, providing evidence that omega-3 intervention has a beneficial effect on liver cell physiology [27]. As such, the FLI may represent an easily available and economic set of biomarkers of relevance for identifying omega-3 sensitive patients and future studies may consider using this tool a priori for patient selection/stratification purposes.

## 5. Conclusions

The current randomized placebo-controlled study supports the use of omega-3 supplementation to increase the omega-3 index in NAFLD patients, significantly greater than that obtained by dietary recommendation alone. This study is therefore in line with recently published research which encourages NAFLD patients to increase their intakes of *n*-3 LC-PUFAs and confirms their beneficial effect [27]. The liver fat content of patients was significantly reduced amongst both placebo and intervention arms, thereby masking any effects omega-3 may have had on the fat content of the liver. Limitations and suggestions for future study design are discussed. In a post-hoc analysis significant placebo adjusted reductions in liver fat were seen in sub-populations with a high FLI score. This economic and easily available test may provide a simple means of identifying omega-3 responsive patients.

## Figures and Tables

**Table 1:** Baseline anthropometric and biochemical variables of participants randomized to placebo and MF4637 groups.

Variables	Placebo ^1^	MF4637 ^2^	*p*-Value *
Age, years	55.1 (10.9)	55.3 (13.3)	0.93
Sex, M/F	44/42	36/45	0.39
Weight, kg	90.1 (18.8)	88.4 (18.4)	0.55
Waist circumference, cm	105.9 (13.1)	106.3 (13.2)	0.87
Hip circumference, cm	110.5 (12.1)	110.9 (11.8)	0.85
Waist to hip ratio	0.96 (0.09)	0.96 (0.08)	0.95
BMI, kg/m^2^	32.4 (5.0)	32.1 (4.8)	0.59
Systolic blood pressure, mm Hg	127.0 (10.7)	128.0 (11.9)	0.64
Diastolic blood pressure, mm Hg	80.3 (7.2)	79.8 (7.6)	0.33
Heart rate, beats/min	74.7 (8.7)	73.2 (9.1)	0.25
Statin use, %	34.9	30.9	0.58
Diabetes, %	39.5	35.0	0.97
Fasting glucose, mg/dL	120.1 (48.5) ^2^	119.4 (38.1) ^3^	0.97
Fasting insulin, µIU/mL	30.2 (41.3)	20.8 (18.2) ^4^	0.04
HbA1c, %	6.5 (1.5) ^5^	6.3 (1.4) ^6^	0.20
Triglycerides, mg/dL	199.1 (123.0) ^7^	192.0 (125.1) ^4^	0.70
BUN, mg/dL	14.7 (4.9) ^7^	15.4 (5.0) ^4^	0.24
Creatinine, mg/dL	0.8 (0.2) ^7^	0.3 (0.2) ^4^	0.83
TSH µIU/mL	1.9 (1.0) ^7^	1.7 (0.9) ^8^	0.17
Hs-CRP, mg/L	6.4 (9.2) ^7^	8.1 (17.5)	0.61
Albumin, g/dL	4.29 (0.3) ^7^	4.29 (0.3) ^4^	0.91
ALT, IU/L	35.6 (24.0)	37.5 (39.0)	0.40
AST, IU/L	25.8 (12.2)	27.1 (20.3)	0.79
ALP, IU/L	81.5 (31.0)	85.5 (45.3)	0.62
GGT, IU/L	47.1 (49.0)	62.2 (151.4)	0.78
Bilirubin, mg/dL	0.5 (0.2)	0.5 (0.3)	0.91

^1^ Data for *n* = 86 participants unless otherwise specified. ^2^ Data for *n* = 81 participants unless otherwise specified. ^3^ Data for *n* = 75 participants. ^4^ Data for *n* = 80 participants. ^5^ Data for *n* = 83 participants. ^6^ Data for *n* = 79 participants. ^7^ Data for *n* = 85 participants. ^8^ Data for *n* = 78 participants. Values expressed as Mean (SD). Abbreviations: ALP, alkaline phosphatase; ALT, alanine aminotransferase; AST, aspartate aminotransferase; BMI, body mass index; BUN, blood urea nitrogen; GGT, gamma-glutamyl transferase; HbA1c, glycated haemoglobin; hs-CRP, high-sensitivity C-reactive protein; TSH, thyroid stimulating hormone. * All statistical tests performed were *t*-tests except for chi-square tests for the following variables: Sex; Statin use and Diabetes, and Wilcoxon two-sample tests for the following variables: Fasting glucose; HbA1c; ALP and GGT.

**Table 2:** Anthropometric and biochemical variables of participants randomized to placebo and MF4637 groups at baseline and study completion.

Variables	Placebo ^1^			MF4637 ^2^			Two-Sample Test for Change from Baseline *p*-Value ^#^
	Baseline	End of Study	*p*-Value *	Baseline	End of Study	*p*-Value *	
Age, years	55.1 (10.9)			55.3 (13.3)			
Sex, M/F	44/42			36/45			
Weight, kg	90.1 (18.8)	89.5 (18.5)	0.84	88.4 (18.4)	89.0 (18.8)	0.082	0.23
Waist circumference, cm	105.9 (13.1)	104.7 (13.9)	0.005	106.3 (13.2)	105.5 (12.2)	0.72	0.18
Hip circumference, cm	110.5 (12.1)	110.0 (11.7)	0.31	110.9 (11.8)	110.4 (11.9)	0.81	0.42
Waist to hip ratio	0.96 (0.1)	0.95 (0.1)	0.081	0.96 (0.08)	0.96 (0.1)	0.66	0.58
BMI, kg/m^2^	32.4 (5.0)	32.3 (4.8)	0.74	32.1 (4.8)	32.3 (5.0)	0.078	0.18
SBP, mm Hg	127.0 (10.7)	128.8 (15.0)	0.23	128.0 (11.9)	126.6 (10.6)	0.29	0.11
DBP, mm Hg	80.3 (7.2)	79.9 (9.9)	0.25	79.8 (7.6)	77.8 (8.4)	0.032	0.53
Heart rate, beats/min	74.7 (8.7)	74.1 (8.2)	0.59	73.2 (9.1)	74.2 (9.8)	0.17	0.17
Statin use, %	34.9			30.9			
Diabetes, %	39.5			35.0			
Fasting glucose, mg/dL	120.1 (48.5) ^3^	125.4 (56.8) ^3^	0.39	119.4 (38.1) ^3^	127.5 (55.2) ^3^	0.0616	0.38
Fasting insulin, µIU/mL	30.2 (41.3) ^4^	30.1 (35.8) ^4^	0.51	20.8 (18.2) ^5^	24.0 (24.3) ^5^	0.21	0.63
HbA1c, %	6.5 (1.5) ^6^	6.6 (1.7) ^6^	0.67	6.3 (1.4) ^7^	6.3 (1.4) ^7^	0.83	0.87
Triglycerides, mg/dL	199.1 (123.0) ^2^	185.7 (118.0) ^2^	0.52	192.0 (125.1) ^5^	157.8 (84.2) ^5^	0.0008	0.053
BUN, mg/dL	14.7 (4.9) ^2^	15.0 (4.5) ^2^	0.88	15.4 (5.0) ^5^	16.2 (4.9) ^5^	0.25	0.46
Creatinine, mg/dL	0.8 (0.2) ^2^	0.82 (0.2) ^2^	0.51	0.3 (0.2) ^5^	0.84 (0.2) ^5^	0.26	0.19
TSH µIU/mL	1.9 (1.0) ^2^	2.5 (2.7) ^2^	0.025	1.7 (0.9) ^7^	1.8 (0.9) ^7^	0.043	0.86
Hs-CRP, mg/L	6.4 (9.2) ^2^	5.5 (5.8) ^2^	0.46	8.1 (17.5) ^8^	6.7 (10.9) ^8^	0.75	0.82
Albumin, g/dL	4.29 (0.3) ^2^	4.3 (0.3) ^2^	0.69	4.29 (0.3) ^5^	4.3 (0.3) ^5^	0.62	0.91
ALT, IU/L	35.6 (24.0)	29.8 (21.2)	0.005	37.5 (39.0)	38.1 (37.7)	0.48	0.015
AST, IU/L	25.8 (12.2)	23.9 (13.5)	0.036	27.1 (20.3)	28.7 (24.6)	0.37	0.036
ALP, IU/L	81.5 (31.0)	76.3 (21.5)	0.01	85.5 (45.3)	83.2 (40.3)	0.09	0.73
GGT, IU/L	47.1 (49.0)	37.1 (32.2)	<.0001	62.2 (151.4)	57.1 (108.7)	0.37	0.058
Bilirubin, mg/dL	0.5 (0.2)	0.47 (0.2)	0.70	0.5 (0.3)	0.5 (0.2)	0.88	0.72

^1^ Data for *n* = 86 participants unless otherwise specified. ^2^ Data for *n* = 81 participants unless otherwise specified. ^3^ Data for *n* = 72 participants. ^4^ Data for *n* = 82 participants. ^5^ Data for *n* = 78 participants. ^6^ Data for *n* = 74 participants. ^7^ Data for *n* = 76 participants. ^8^ Data for *n* = 79 participants. Values are expressed as Mean (SD). Abbreviations: ALP, alkaline phosphatase; ALT, alanine aminotransferase; AST, aspartate aminotransferase; BMI, body mass index; BUN, blood urea nitrogen; DBP, diastolic blood pressure; GGT, gamma-glutamyl transferase; HbA1c, glycated haemoglobin; hs-CRP, high-sensitivity C-reactive protein; SBP, systolic blood pressure.* Within-group differences were assessed using paired *t*-tests or Wilcoxon signed rank tests. ^#^ Intergroup differences were assessed using two-sample *t*-tests or two-sample Wilcoxon tests.

**Table 3:** RBC fatty acid content at baseline and after 12- and 24-week intervention with placebo or MF4637.

	Placebo (*n* = 86)				MF4637 (*n* = 81)				*p*-Value ^1^
	Baseline	T = 12 weeks	T = 24 weeks	Change from Baseline	Baseline	T = 12 weeks	T = 24 weeks	Change from Baseline	
RBC omega-3 index, %	4.9 (1.2)	5.8 (1.3)	5.3 (1.1)	0.4 (1.0)	4.8 (1.1)	8.7 (2.3)	8.0 (2.6)	3.2 (2.7)	<0.0001
RBC EPA + DHA, µg/mL	32.3 (26.4)	34.9 (21.0)	33.1 (20.5)	1.2 (14.9)	29.6 (17.5)	51.5 (38.9)	52.9 (40.7)	21.2 (28.7)	<0.0001
RBC EPA, %	0.54 (0.3)	0.54 (0.2)	0.54 (0.2)	0.002 (0.3)	0.53 (0.2)	1.6 (0.9)	1.4 (0.9)	0.9 (1.0)	<0.0001
RBC EPA, µg/mL	3.8 (6.6)	3.7 (2.6)	4.1 (5.7)	0.4 (7.0)	3.0 (2.6)	10.4 (10.7)	10.6 (12.0)	7.1 (10.5)	<0.0001
RBC DHA, %	4.3 (1.1)	5.2 (1.2)	4.8 (1.0)	0.4 (0.9)	4.3 (1.0)	7.1 (1.5)	6.6 (1.8)	2.3 (1.9)	<0.0001
RBC DHA, µg/mL	28.5 (21.0)	31.2 (18.6)	29.0 (16.2)	0.7 (10.7)	26.6 (15.3)	41.0 (28.8)	42.4 (29.8)	14.1 (19.5)	<0.0001
RBC omega-6: omega-3	4.9 (1.1)	4.5 (0.9)	4.7 (0.8)	−0.2 (0.7)	4.9 (1.2)	3.0 (0.9)	3.3 (1.5)	−1.6 (1.8)	<0.0001

^1^*p*-value is for the mean percentage change from baseline to 24 weeks between placebo and MF4637 groups using ANCOVA. Values are expressed as Mean (SD). Abbreviations: RBC, red blood cell; DHA, docosahexaenoic acid; EPA, eicosapentaenoic acid; T: Time.

**Table 4:** MRI-PDFF liver fat percentage at baseline and after 24-week intervention with placebo or MF4637.

	Placebo ^1^				MF4637 ^1^				*p*-Value
	T = 0 weeks	T = 24 weeks	Change from Baseline		T = 0 weeks	T = 24 weeks	Change from Baseline		
			Absolute	Relative			Absolute	Relative	
Liver fat, %	17.4 (10.4)	12.6 (8.0)	−4.4 (6.9)	−27.6	14.4 (10.1)	10.7 (7.6)	−2.8 (5.8)	−25.7	0.1838

^1^ As assessed for modified ITT population (Placebo, *n* = 60; MF4637, *n* = 60). Values expressed as Mean (SD); T: Time.

**Table 5:** Change in MRI-PDFF liver fat percentage after 24-week intervention with MF4637 stratified by baseline FLI score.

		Baseline HFF Mean (SD)	Study End HFF Mean (SD)	Change in MRI-PDFF Liver Fat Percentage (%)			
				Absolute Change	*p*-Value ^1^	Relative Change	*p*-Value ^1^
FLI <30 ^2^	Placebo	15.6 (11.2)	11.2 (7.8)	2.25 (1.39)	0.11	2.7 (11.3)	0.81
	Omega-3	12.5 (8.6)	10.3 (7.7)				
FLI ≥30 ^3^	Placebo	20.6 (9.4)	16.0 (7.9)	−2.47 (2.53)	0.34	−9.3 (15.3)	0.55
	Omega-3	19.3 (11.1)	13.5 (7.0)				
FLI ≥40 ^4^	Placebo	20.2 (9.5)	16.3 (8.4)	−7.45 (2.81)	0.02	−44.1 (14.6)	0.009
	Omega-3	20.7 (10.4)	10.6 (5.2)				

^1^*p*-value is for the mean percentage change from baseline to 24 weeks (placebo corrected) using ANCOVA. ^2^ Data for *n* = 89 participants (*n* = 43 placebo; *n* = 46 MF4637). ^3^ Data for *n* = 28 participants (*n* = 16 placebo; *n* = 12 MF4637).^4^ Data for *n* = 17 participants (*n* = 12 placebo; *n* = 5 MF4637). Values expressed as Mean (SD). Abbreviations: FLI, Fatty Liver Index; HFF, Hepatic Fat Fraction.

## Supplementary Materials

The following are available online at http://www.mdpi.com/2072-6643/10/8/1126/s1.
